# Characterization of MUDENG, a novel anti-apoptotic protein

**DOI:** 10.1038/oncsis.2016.30

**Published:** 2016-05-02

**Authors:** J-H Choi, J-B Lim, D D Wickramanayake, Y Wagley, J Kim, H-C Lee, H G Seo, T-H Kim, J-W Oh

**Affiliations:** 1 Department of Animal Biotechnology, College of Animal Bioscience and technology/Animal Resources Research Center, Konkuk University, Seoul, Korea; 2 Department of Genomics, Wide River Institute of Immunology, Seoul National University, Gangwon, Korea; 3 Department of Biochemistry, Chosun University School of Medicine, 309 Pilmoondaero, Gwangju, Korea

## Abstract

*MUDENG* (*Mu-2-related death-inducing gene*, *MuD*) is revealed to be involved in cell death signaling. Astrocytes, the major glial cell type in the central nervous system, are a source of brain tumors. In this study, we examined MuD expression and function in human astroglioma cells. Stimulation of U251-MG cells with tumor necrosis factor-related apoptosis-inducing ligand (TRAIL) resulted in a 40% decrease in cell viability and a 33% decrease in MuD protein levels, although not in *MuD* mRNA levels. To study the functional relevance of MuD expression, stable transfectants expressing high levels of MuD were generated. Stimulation of these transfectants with TRAIL resulted in enhanced cell survival (77% for stable and 46% for control transfectants). Depletion of *MuD* led to a marked reduction upon TRAIL stimulation in cell viability (22% in *MuD*-depleted cells and 54% in control cells). In addition, we observed that *MuD* depletion increased the susceptibility of the cells to TRAIL by enhancing the cleavage of caspase-3/-9 and BH3-interacting domain death agonist (Bid). A unique 25-kDa fragment of B-cell lymphoma 2 (Bcl-2) lacking BH4 was observed 60–180 min post TRAIL treatment in *MuD*-depleted cells, suggesting that Bcl-2 is converted from its anti-apoptotic form to the truncated pro-apoptotic form. Importantly, the TRAIL-mediated decrease in cell viability in *MuD*-depleted cells was abrogated upon *Bid* depletion, indicating that the role of MuD in apoptotic signaling takes place at the Bid and Bcl-2 junction. MuD localizes predominantly in the endoplasmic reticulum and partly in the mitochondria and its amounts are reduced 6 h post TRAIL stimulation, presumably via caspase-3-mediated MuD cleavage. Collectively, these results suggest that MuD, a novel signaling protein, not only possesses an anti-apoptotic function but may also constitute an important target for the design of ideal candidates for combinatorial treatment strategies for glioma cells.

## Introduction

Glioblastoma multiforme (GBM) is a heterogeneous tumor, containing multiple genetically aberrant clones; it is the most common and aggressive malignant form of astrocytoma with a median survival of ~12–15 months.^1, 2^ In spite of improved surgical techniques and advanced radio/chemotherapy, the survival time of GBM patients has not been extended with any actual beneficial effect.^3, 4^ Recently, a promising therapeutic approach was introduced for GBM; selective induction of apoptosis using the pro-apoptotic cytokine tumor necrosis factor-related apoptosis-inducing ligand (TRAIL). Recombinant soluble TRAIL exhibits strong tumoricidal activity against GBM cells with no or minimal toxicity against normal cells.^5^ However, recent studies indicate that no single therapeutic agent, including TRAIL, is likely to be effective enough.^3, 6^ Therefore, the anti-GBM activity of TRAIL, an ideal candidate for combinatorial strategies, was combined with a variety of conventional or novel targeted therapies to achieve synergistic enhancement of TRAIL activity.^5, 6^

Apoptosis is necessary to maintain cell homeostasis in the body. It is generally initiated via two pathways; the extrinsic pathway, mediated by death receptors belonging to the tumor necrosis factor-receptor superfamily such as TRAIL-R1/-R2,^7^ and the intrinsic pathway, triggered in response to cellular stress and DNA damage and involving the release of pro-apoptotic factors from the mitochondria.^8^

TRAIL-induced TRAIL-R activation leads to the formation of the death-inducing signaling complex via recruitment of the adapter protein Fas-associated death domain and caspase-8. The formation of death-inducing signaling complex enables auto-activation of the recruited caspases. Following the activation of caspases-8/-10, the apoptotic signaling cascade targets caspase-3 for proteolytic cleavage; activated caspase-3 in turn cleaves numerous cellular proteins, resulting in the classical features of apoptosis. B-cell lymphoma 2 (Bcl-2) Homology (BH) 3-interacting domain death agonist (Bid) is also cleaved by active caspase-8, generating truncated Bid (tBid). tBid initiates the intrinsic pathway of apoptosis by binding to Bcl-2-associated X (BAX) and Bcl-2 homologous antagonist/killer, thus amplifying the death-receptor apoptotic signal.^9, 10^ Depending on cell type, proteolytic cleavage of Bid may function as a primary mechanism of TRAIL-induced apoptosis or may serve to amplify the apoptotic response by mediating the simultaneous activation of the extrinsic and intrinsic apoptotic pathways.^11^

*MUDENG* (*Mu-2-related death-inducing gene* (*MuD*)) is a novel gene suggested through a screen for genes associated with Fas-mediated apoptosis.^12^ Further study has revealed that the *MuD* gene encodes a 490 amino acid protein with a predicted size of 54.7 kDa. In addition, analysis of MuD shows that it contains a Mu (μ) homology domain found in adapter proteins that have important roles in intracellular trafficking pathways.^13^ MuD was initially known to be involved in cell death in cytotoxic T cells.^13^ Hirst *et al.*,^14^ recently reported that C14orf108 is a component of adapter protein-5, a newly identified adapter protein complex involved in endosomal trafficking. Surprisingly, *C14orf108* was shown to be the same gene as *MuD.*

In this study, we investigated the function of MuD in TRAIL-mediated apoptotic signaling; specifically, the range of intracellular events involving MuD occurring in response to TRAIL binding to its cognate receptor in human astroglioma cells.

## Results and discussion

### MuD domain structure and generation of a monoclonal antibody

**Figure 1:**
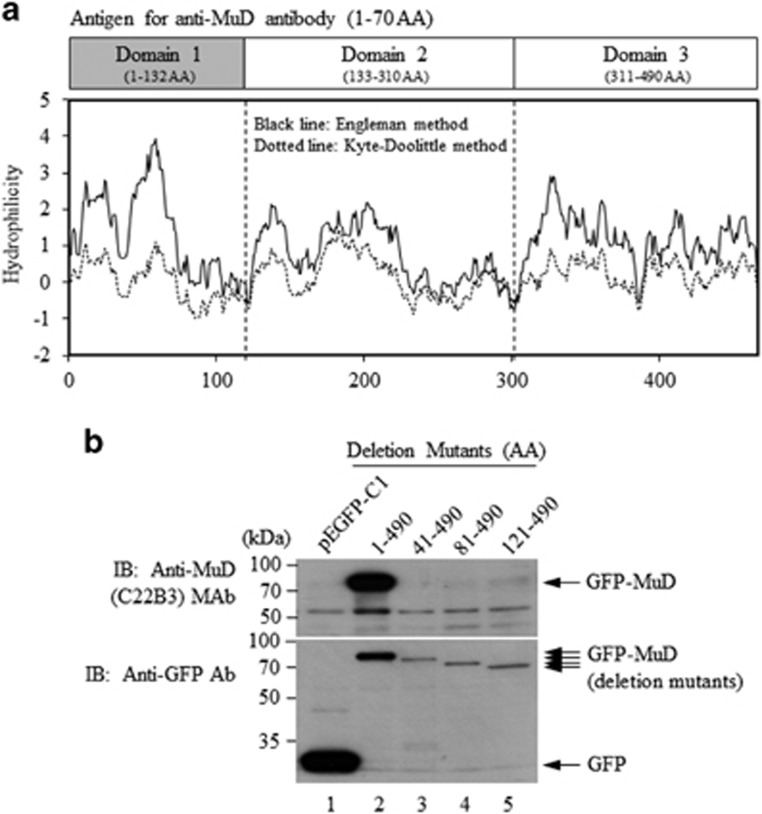
MuD domain analysis and generation of a MuD monoclonal antibody (MAb)-recognizing domain 1 of MuD. (**a**) The hydrophilicity score was calculated based on the methods of Goldman, Engelman and Steitz (GES)^25^ and Kyte–Doolittle (KD)^26^ using the CLC Genomics Workbench program (http://www.clcbio.com/products/clc-genomics-workbench/), which was also used for calculating the isoelectric point (pI). The hydrophilicity of the MuD amino acid sequence (NCBI Reference Sequence: NM_018229.3) was evaluated using the GES (solid black line) and KD (dotted line) methods;^25, 26^ two hydrophilic regions (domain 1, pI=6.82; domain 3, pI=8.34) surround a non-hydrophilic region (domain 2, pI=4.32). (**b**) Domain 1 of MuD was expressed in *Escherichia coli* BL21 (DE3) using pET23dw-His-MuD and then purified using His-bind resin^27^ (Novagen, San Diego, CA, USA). The purified MuD protein was used to generate MAb; immunization, cell fusion and selection of hybridoma clones, and production and purification of the MAb were performed according to the standard procedures.^28^ A mouse MuD MAb (C22B3) produced from one of the hybridomas was used for the experiments. Green fluorescent protein (GFP)-tagged MuD deletion mutants (pEGFPC1-MuD AA 1-490; AA 41−490; AA 81−490; AA 121−490) were generated by PCR. All cDNA constructs were verified by DNA sequencing and expressed in HCT-116 cells obtained from the American Type Culture Collection (ATCC; Manassas, VA, USA) by transient transfection using Lipofectamine2000 (Invitrogen, Gaithersburg, MD, USA). Anti-GFP was purchased from Santa Cruz (Dallas, TX, USA) and horseradish peroxidase-conjugated goat anti-mouse IgGs were obtained from Jackson ImmunoResearch Laboratories (West Groove, PA, USA).

MuD is highly conserved in both mammalian and non-mammalian species.^13^ In particular, human and mouse MuD are 85% identical, whereas human and mouse Bid are 63% identical. MuD is a hydrophilic protein with a mostly hydrophilic N-terminus (designated domain 1; Figure 1a). In addition, MuD contains eight conserved cysteine residues; tertiary structure modeling predicts one in domain 1 (Cys^76^), three in domain 2 (Cys^173^, Cys^246^, and Cys^278^) and four in domain 3 (Cys^318^, Cys^361^, Cys^429^ and Cys^451^). In addition, MuD contains a highly conserved putative glycosylation site at Asn^259^-X^260^-Ser^261^; however, the glycosylation status of MuD remains to be elucidated.

The mouse anti-MuD monoclonal antibody, C22B3, was generated against amino acids 1–70 of MuD domain 1 (Figure 1a), and was characterized for its ability to bind full-length MuD and truncated mutants. The C22B3 monoclonal antibody was capable of detecting full-length but not the deletion mutants (Figure 1b), suggesting that the MuD epitope to which C22B3 monoclonal antibody binds is located within the amino acids 1–40 of domain 1.

### MuD protein expression is correlated with cell viability in TRAIL-treated astroglioma cells

**Figure 2:**
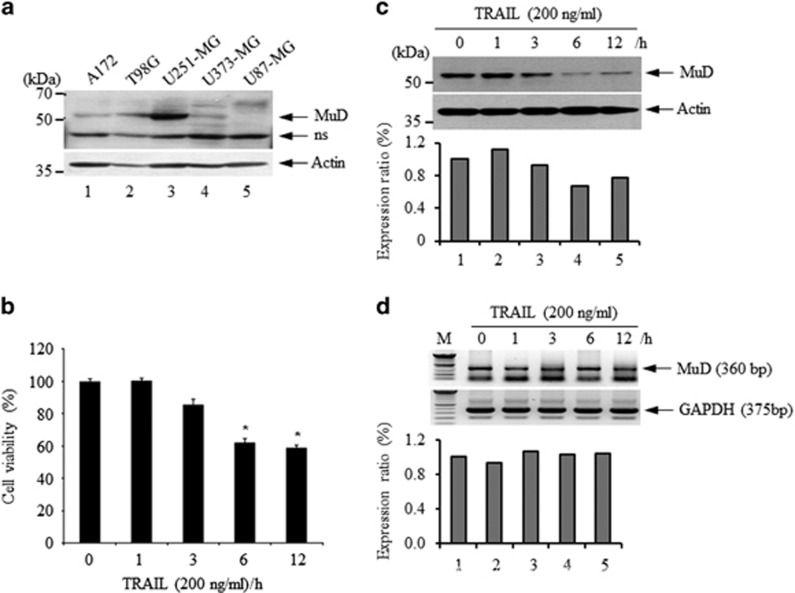
Viability and the expression level of the MuD protein, but not *MuD* mRNA, decreased following TRAIL treatment. U251-MG, U373-MG and U87-MG cells were obtained from Dr Benveniste EN (University of Alabama at Birmingham, Birmingham, AL, USA). A172 and T98G cells were obtained from Dr Lee JH (Department of Biochemistry, College of Medicine, The Catholic University of Korea, Seoul, Korea). (**a**) Cell lysates (30 μg) obtained from each cells were separated by 10% SDS-polyacrylamide gel electrophoresis (SDS-PAGE) then transferred onto a polyvinylidene fluoride (PVDF) membrane. The pattern of MuD protein expression was analyzed by western blot using C22B3 monoclonal antibody (MAb); anti-β-actin (Santa Cruz) was used to analyze β-actin as a loading control. (**b**) Cells (2 × 10^4^ cells/well) grown in 96-well plates were treated for the indicated time (0–12 h) with 200 ng/ml recombinant TRAIL (Peprotech, Rocky Hill, NJ, USA). Cell viability was measured using a colorimetric assay for 96-well plates with 2-(4-iodophenyl)-3-(4-nitrophenyl)-5-(2,4-disulfophenyl)-2H-tetrazolium monosodium salt (WST-1) reagent (Roche Applied Science, Mannheim, Germany). Absorbance was determined at 450 nm using a microplate reader (Bio-Tek, Winooski, VT, USA). Absorbance was directly proportional to the number of viable cells, because the tetrazolium salts in the WST-1 are cleaved to formazan by mitochondrial dehydrogenases in the cells. The level of significance for comparisons between samples was determined using Student's *t*-test distribution. The results presented are the mean±s.d. of three experiments (significant versus control, *P<0.05). (**c**) Cells (3 × 10^5^ cells/well) grown in 6-well plates were treated with TRAIL (200 ng/ml) for the indicated times (0–12 h). Cell lysates were separated by 10% SDS-PAGE then transferred onto a PVDF membrane. The pattern of MuD protein expression was analyzed using C22B3 MAb; β-actin served as a loading control. (**d**) For RT–PCR analysis, total RNA was extracted from prepared U251-MG cells using TRIzol reagent (Ambion, Carlsbad, CA, USA). First-strand DNA synthesis was conducted with oligo (dT) and AccuPower CycleScript RT PreMix kit (BIONEER, Daejeon, Korea) followed by PCR. The amplification program was as follows: 1 cycle of 96 °C/5 min; 35 cycles of 96°C/30 s, 58 °C/30 s, 72 °C/30 s and 1 cycle of 72 °C/7 min. PCR products were detected on a 1% agarose gel with ethidium bromide and analyzed by ChemiImager 5500 (Alpha Innotech, San Leandro, CA, USA). The following primers were used: *MuD* sense: 5′-GGGATACAGGATTTTCTT-3′ *MuD* anti-sense: 5′-GAGACTCAAGCTGATGGT-3′ *hGAPDH* sense: 5′-GTCTTCACCCCATGGAGAAGG-3′ *hGAPDH* anti-sense: 5′-CGTTCAGCTCTGGGATGACCTTG-3′. NS, non-specific.

First, to examine the expression level of MuD protein in the cells, we tested various human malignant glioma cell lines by western blot. As shown in Figure 2a, constitutive expression of MuD protein was strongly detected in U251-MG cells, and weakly detected in A172, T98G and U373-MG cells. On the contrary, MuD expression was hardly detected in U87-MG cells (Figure 2a), suggesting that constitutive expression of MuD protein is quite different from each other, although all of them are designated as glioma cells. Subsequently, as MuD was identified during a screen for genes associated with death-receptor-mediated apoptosis,^12^ we examined whether the expression of MuD in astroglioma cells could be modulated by a pro-apoptotic cytokine known to induce cell death. To do this, U251-MG cells were treated with TRAIL for various durations of time (1–12 h) and cell viability was then analyzed. As shown in Figure 2b, the effect of TRAIL on cell viability became noticeable at 3 h, and ~40% decrease was observed at 12 h. To examine if MuD protein expression is altered, expression kinetics in TRAIL-treated cells were determined by western blot analysis. As shown in Figure 2c, constitutive expression of MuD protein was detected (lane 1). Interestingly, the amount of MuD protein began to decrease at 3 h (lane 3), reaching maximal reduction at 6 h (lane 4, 33% inhibition), a level that was sustained for 12 h (lane 5). We also tested if MuD expression is altered in T98G cells. Amount of MuD protein in TRAIL-treated T98G cells began to decrease at 6 h and sustained for 24 h (Supplementary Figure S1). To test whether MuD mRNA levels also varied, expression kinetics were examined by reverse transcription–polymerase chain reaction. As shown in Figure 2d, MuD mRNA was constitutively expressed (lane 1) and its level was not influenced by TRAIL (lanes 2–5). Previously, we suggested that MuD may be a novel caspase-3 substrate in response to TRAIL stimulation in Jurkat T cells.^15^ Therefore, TRAIL-activated caspase-3 proteolytic cleavage of MuD could provide a possible explanation for the alteration in amounts of MuD protein.

### Differences in cell viability in response to TRAIL in stable MuD transfectants and MuD-depleted cells

**Figure 3:**
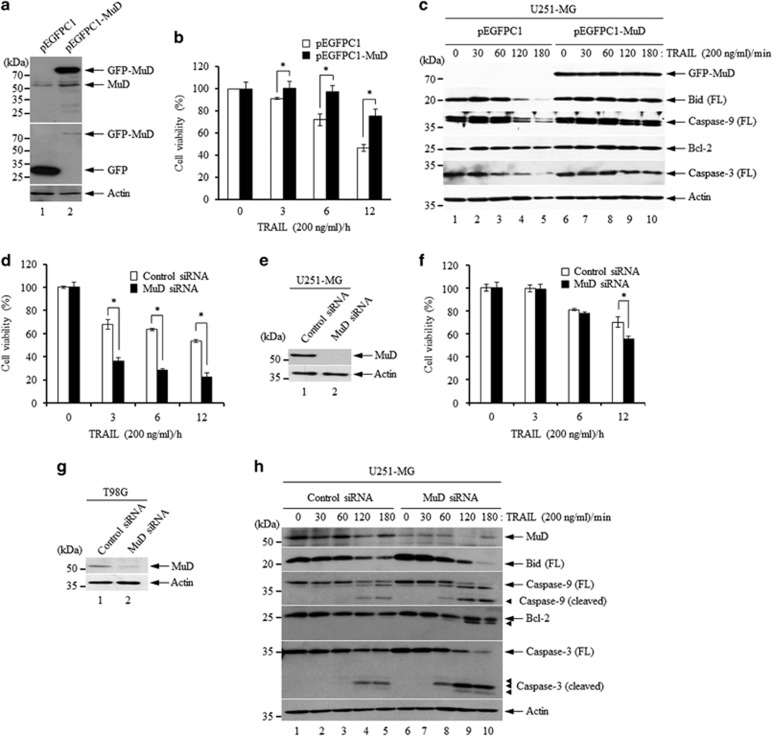
MuD exerts an inhibitory effect on TRAIL-induced sensitivity to death stimuli. (**a**) U251-MG cells stably expressing GFP alone (pEGFPC1) and GFP-MuD (pEGFPC1-MuD) were generated by transfection using Lipofectamine2000 and selected on G418 sulfate (200 μg/ml; Invitrogen). Amount of MuD was analyzed by western blot using C22B3 MAb and anti-GFP Ab (Santa Cruz). The blot was re-probed with anti-β-actin. (**b**) pEGFPC1 (white) and pEGFPC1-MuD (black) stable transfectants were stimulated by TRAIL for the indicated time periods (0–12 h). Cell viability was measured using WST-1. Data presented are the mean±s.d. of three experiments (significant versus control, *P<0.05). (**c**) pEGFPC1 and pEGFPC1-MuD transfectants were seeded at a density of 3 × 10^6^ cells in 100-mm culture dishes and incubated overnight. Cells were then treated with TRAIL (200 ng/ml) for the indicated times (0–180 min). Cell lysates were separated on 10% SDS-PAGE and analyzed by western blot with the following antibodies; C22B3 MAb, anti-Bid, anti-caspase-3 (Cell Signaling Technology, Danvers, MA, USA), anti-caspase-9, anti-Bcl-2 (Santa Cruz) and β-actin as a loading control. (**d**, **f**) *MuD* siRNA (sense strand: 5′-GAGCAAGUUAUGUGCCUGUdTdT-3′ anti-sense strand: 5′-ACAGGCACAUAACUUGCUC-3′), a target-specific 19-nt siRNA designed to silence the gene expression and control siRNA (sense strand: 5′-CCUACGCCACCAAUUUCGU-3′ anti-sense strand: 5′-ACGAAAUUG GUGGCGUAGG-3′) were purchased from BIONEER. U251-MG (**d**) and T98G cells (**f**) were seeded at a density of 3 × 10^5^ cells in 6-well plates, respectively. Twelve hours after seeding, cells were transfected with control and *MuD* siRNA duplexes (100 nM) using Lipofectamine2000. Following 36 h incubation, 2 × 10^4^ cells were then re-seeded into 96-wells, incubated for 12 h, and then treated with TRAIL (200 ng/ml) for the indicated time periods (0–12 h). Cell viability was measured using WST-1. Data presented are the mean±s.d. of three experiments (significant versus control, *P<0.05). (**e**, **g**) Each cell from the experiment presented in **d** and **f** were lysed, subjected to 10% SDS-PAGE, and amount of MuD was analyzed by western blot using C22B3 MAb. The blot was re-probed with anti-β-actin. (**h**) U251-MG cells were seeded at a density of 3 × 10^6^ cells in 100-mm cell culture dishes. Following overnight incubation, the cells were transfected with control and *MuD* siRNA duplexes (100 nM) using Lipofectamine2000, respectively. Subsequent to 36 h incubation, the cells were re-seeded into 6 wells (3 × 10^5^ cells/well), incubated for an additional 24 h, and then stimulated with TRAIL for the indicated times (0–180 min). The expression patterns of MuD and the caspases were detected by western blot analysis with the following antibodies; C22B3 MAb, anti-Bid, anti-caspase-9, anti-caspase-3, anti-Bcl-2 and β-actin as a loading control. FL, full length.

In order to discern the functional consequence of MuD expression in U251-MG cells, stable transfectants overexpressing *MuD* were generated; the transfectants for further experiments were selected based on high MuD expression as determined by western blot analysis (Figure 3a). Cell viability of the stable transfectants upon TRAIL stimulation was analyzed by WST-1 assay. As shown in Figure 3b, difference of cell viability was evident between the stable and control transfectants at 3 h post TRAIL treatment, reaching maximum at 12 h (77% for stable and 46% for control transfectants). Simultaneously, we analyzed proteolytic cleavage of caspase-3/-9, Bid, Bcl-2 and MuD in stable and control transfectants following TRAIL treatment for various times (0–180 min). Proteolytic cleavages of caspase-3/-9 and Bid were delayed in stable transfectants (120–180 min, lanes 9 and 10) compared with that in the control (120–180 min, lanes 4 and 5), whereas there was no observed marked difference in Bcl-2 molecules between the two groups (Figure 3c). These results suggest that the anti-apoptotic function of MuD may enable cell resistance to death stimuli.

We further determined the functional properties of MuD through gene depletion by small interfering RNA (siRNA) in U251-MG and T98G cells. As shown in Figure 3d, *MuD* depletion resulted in a significant reduction in the cell viability of U251-MG cells following TRAIL treatment for 12 h; TRAIL treatment in control cells resulted in ~54% viability, whereas in *MuD*-depleted cells it led to ~22% cell viability. *MuD* depletion by siRNA transfection was confirmed by western blot analysis (Figure 3e, lane 2). In case of T98G cells, TRAIL treatment for 12 h in control cells showed ~70% viability, whereas ~55% cell viability in *MuD*-depleted cells was observed (Figures 3f and g, lane 2). These results indicate that *MuD* depletion causes these cells to undergo massive cell death following TRAIL treatment, consistent with the results obtained for the stable transfectants overexpressing *MuD* (Figure 3b). On the basis of these results, we hypothesize that high intracellular levels of MuD inside cell may, at least in part, contribute to aberrant cells escaping the normal tumor surveillance system, possibly via the inhibition of apoptotic pathway; these cells can then propagate and form additional tumors.

Next, we examined the dynamics of key components involved in the extrinsic apoptosis pathway in the absence of *MuD*. U251-MG cells were transfected with control siRNA or *MuD* siRNA and then treated with TRAIL for various times (0–180 min). As shown in Figure 3h, although the cleavage pattern of caspase-3/-9, Bid and Bcl-2 was comparable in both the groups following TRAIL treatment, proteolytic cleavage of these molecules was initiated earlier in *MuD*-depleted cells (60 min, lane 8) compared with that in the control cells (120 min, lane 4). In particular, a unique 25-kDa fragment of Bcl-2 was detected 60–180 min following TRAIL treatment in *MuD-*depleted cells (lanes 8–10) but not in control cells (lanes 4–5). Recent studies have suggested that the Bcl-2 protein is cleaved into a 23-kDa fragment as the result of several stimuli such as cytokine, photochemical, cisplatin and oxidative stress.^16, 17, 18^ However, we could not observe a 23-kDa cleaved fragment of Bcl-2 in *MuD*-depleted cells. Coincident with our results, Ling *et al.*^19^ reported the presence of a 25-kDa fragment of Bcl-2-lacking the BH4 domain following bortezomib treatment, resulting in cell growth arrest and apoptosis. In addition, a growing evidence indicates that overexpression of a Bcl-2-lacking BH4 results in growth reduction of tumor cell through apoptotic and autophagic process caused by the impairment of Bcl-2 homo/heterodimer binding with BAX or Beclin,^20, 21^ indicating the importance of the BH4 domain for tumor cell survival. Therefore, a possible scenario is that the formation of 25-kDa Bcl-2 fragments by TRAIL in *MuD*-depleted cells converts Bcl-2 from its anti-apoptotic form to the truncated pro-apoptotic form (reviewed by Akl *et al.*^22^), elucidating the functional role of MuD in the regulation of Bcl-2 activity in TRAIL-induced apoptotic signaling. Also, the complete processing of caspase-9 occurred subsequent to the appearance of the 25-kDa fragment of Bcl-2, suggesting that Bcl-2 cleavage may occur upstream of mitochondrial membrane permeabilization and apoptosome formation in *MuD*-depleted cells.

### Bid activation may occur prior to MuD degradation induced by death stimuli

**Figure 4:**
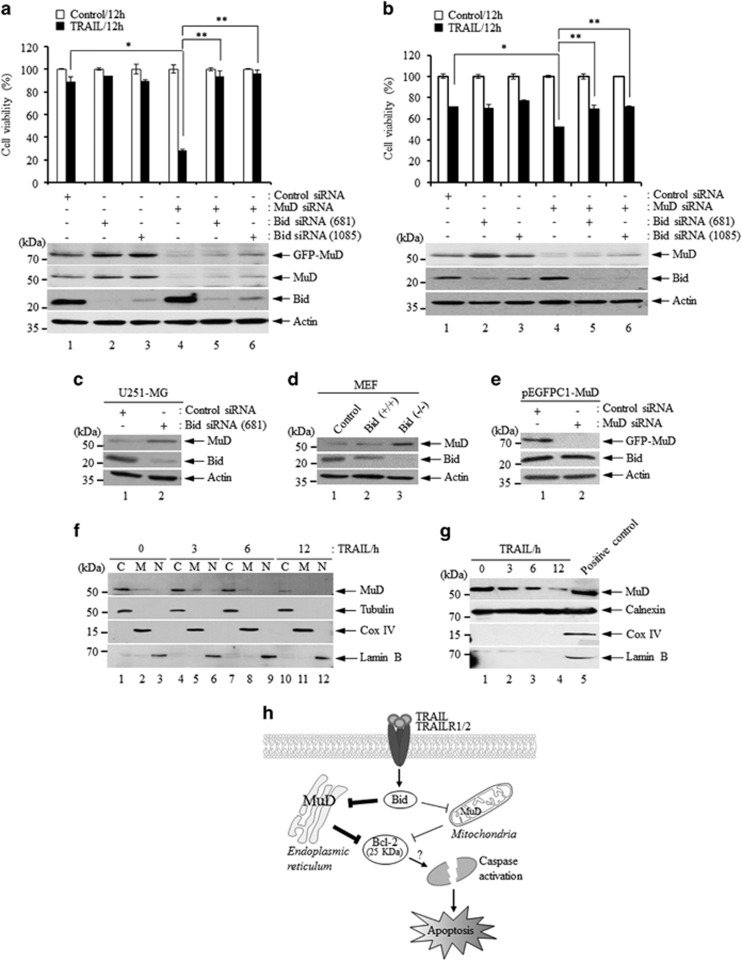
Bid neutralizes MuD function in TRAIL-mediated apoptotic signaling. (**a**, **b**) *Bid* siRNA1 (#681, sense strand: 5′-GGCAGAUUCUGAAAGUCAATT-3′ anti-sense strand: 5′-UUGACUUUCAGAAUCUGCCTT-3′) and *Bid* siRNA2 (#1085, sense strand: 5′-CGAUGUGGUCACAGCUGUATT-3′ anti-sense strand: 5′-UACAGCUGUGACCACAUCGTT-3′) target-specific 21-nt siRNAs designed to silence gene expression were obtained from BIONEER. Control and *MuD* siRNA were used as described in Figures 3d and f. pEGFPC1-MuD transfectants and T98G cells were seeded at a density of 3 × 10^5^ cells in 6-well plates, respectively, and transfected with control, *MuD*, and *Bid* siRNA duplexes and *MuD* plus *Bid* siRNA using Lipofectamine2000. Following 36 h incubation, 2 × 10^4^ cells were re-seeded into 96-wells, incubated for 12 h, and then treated with TRAIL (200 ng/ml) for an additional 12 h. Cell viability was measured using WST-1. Data presented are the mean±s.d. of three experiments (significant versus control, *P<0.05, **P<0.01). The rest of each cell were lysed, subjected to 12% SDS-PAGE, and the patterns of MuD expression were analyzed by western blot using C22B3 MAb (top panel) and anti-Bid Ab (middle panel). The blot was re-probed with anti-β-actin (bottom panel). (**c**) U251-MG cells were seeded at a density of 3 × 10^5^ cells in 6-well plates. The cells were transfected with control and *Bid* siRNA duplexes (#681), respectively, using Lipofectamine2000. Following 36 h incubation, the cells were lysed, subjected to 12% SDS-PAGE, and amount of MuD and Bid was analyzed by western blot using C22B3 MAb (top panel) and anti-Bid Ab (middle panel). The blot was re-probed with anti-β-actin (bottom panel). (**d**) SV-40-transformed (control), *Bid*^+/+^ and *Bid*^−/−^ mouse embryonic fibroblast (MEF) lysates were subjected to 12% SDS-PAGE and analyzed by western blot using C22B3 MAb (top panel) and anti-Bid Ab (middle panel). The blot was re-probed with anti-β-actin (bottom panel). (**e**) pEGFPC1-MuD transfectants were seeded at a density of 3 × 10^5^ cells in 6-well plates and transfected with control and *MuD* siRNA duplexes using Lipofectamine2000. Subsequent to 36 h incubation, cells were lysed and subjected to 12% SDS-PAGE. Amount of MuD and Bid was analyzed by western blot. (**f**) U251-MG cells were grown to 80% confluence in 100-mm culture dishes and then stimulated with TRAIL (200 ng/ml) for the indicated times (0–12 h). The cytoplasmic, mitochondrial and nuclear fractions were isolated from U251-MG cells using a cell fractionation kit (ab109719, Abcam, Cambridge, MA, USA) according to the manufacturer's instructions. Fractions were subjected to 12% SDS-PAGE, and analyzed by western blot using C22B3 MAb to determine the subcellular localization of MuD. The blots were re-probed with anti-Cox IV, anti-tubulin (Abcam) and anti-lamin B (GeneTex, Irvine, CA, USA), respectively. (**g**) The microsomal fraction was isolated from the cultured cells after TRAIL treatment (200 ng/ml) for 0–12 h using an ER isolation kit according to the manufacturer's instructions (ER0100, Sigma, Saint Louis, MO, USA). Sample lysates were analyzed by 12% SDS-PAGE followed by western blot using C22B3 MAb for MuD detection. The blots were re-probed with anti-calnexin (Abcam), anti-Cox IV and anti-lamin B, respectively. (**h**) Proposed mechanism underlying the role of MuD in TRAIL-mediated death signaling. MuD resides at the ER and mitochondria regulates apoptotic signaling induced by the formation of TRAIL/TRAIL-R complex. Bid may be essential for the regulation of MuD expression and function. In addition, the formation of 25-kDa Bcl-2 fragments by TRAIL stimulation which converts Bcl-2 from its anti-apoptotic form to the truncated pro-apoptotic form is inhibited by MuD, affecting regulation of caspase activation which subsequently leads to apoptosis.

The proteolytic cleavage of Bid may serve to amplify the apoptotic response by mediating the activation of the extrinsic apoptotic pathway.^11^ In addition, the apoptotic pathway for mitochondrial cytochrome c release and apoptosome formation following apoptotic stimulus requires Bid.^23, 24^ To determine whether Bid and MuD cooperatively affect MuD activity following death stimuli, the cell viability of stable U251-MG transfectants and T98G cells was analyzed following transfection with *Bid* siRNA (#681 and #1085), *MuD* siRNA or both. As shown in Figure 4a, following 12 h of TRAIL treatment, cell viability of stable U251-MG transfectants was decreased 72% after *MuD* depletion (lane 4), whereas the reduction in the cell viability in *Bid*-depleted cells (#681 or #1085) was minimal (9% decrease, lanes 2–3), compared with that in control siRNA cells. The minimal effect in *Bid*-depleted cells could be due to the presence of an active caspase-3 pathway through caspase-8/-10. Importantly, the decreased rate of cell viability observed in *MuD*-depleted cells (lane 4) was abrogated upon simultaneous *Bid* depletion (#681 or #1085; lanes 5–6). Endogenous MuD protein expression in stable U251-MG transfectants was increased after *Bid* depletion (#681 or #1085; lanes 2–3) compared with control siRNA cells (lane 1), and remained highly even under the condition of both depletion (MuD plus Bid (#1085), lane 6) when compared with MuD alone depletion (lane 4). Each MuD and Bid protein expression was confirmed by western blot analysis (Figure 4a, bottom). Although, in case of T98G cells similar patterns were observed along with stable U251-MG transfectants, there was no marked difference as observed in stable U251-MG transfectants (Figure 4b). We guess that the reason for this is the differences of constitutive MuD expression. To further elucidate these findings, MuD expression was examined in U251-MG following *Bid* and control siRNA transfection. As shown in Figure 4c, the amount of MuD protein was increased in *Bid*-depleted cells (lane 2) compared with control cells (lane 1). This phenomenon was also observed in *Bid*^−/−^ mouse embryonic fibroblasts cells (Figure 4d, lane 3). In contrast, Bid expression was not altered in stable U251-MG transfectants after *MuD* depletion (Figure 4e, lane 2). These results indicate that Bid cleavage may occur prior to MuD activation and have a key role in the functional properties of MuD in TRAIL-mediated apoptotic signaling.

Next, we studied the subcellular localization of MuD in the cells. U251-MG were stimulated with TRAIL for the indicated times (0–12 h) and the presence of MuD was then determined in the cytoplasmic, mitochondrial and nuclear fractions by western blot analysis. As shown in Figure 4f, MuD was predominantly localized in the cytoplasmic compartment, partly in the mitochondria, but was absent in the nucleus (lanes 1–3). Amount of MuD began to decrease in both compartments at 6–12 h following TRAIL treatment (lanes 7–8 and 10–11), presumably due to caspase-3-mediated MuD cleavage.^15^ To further clarify MuD localization in cytoplasmic compartment, the microsomal fraction was isolated from the cells following TRAIL treatment using an endoplasmic reticulum (ER) isolation kit. As shown in Figure 4g, we observed that amount of MuD is highly localized in the ER compartments (lane 1), and began to diminish upon TRAIL stimulation (lanes 2–4), suggesting ER's importance for MuD localization and function.

In conclusion, to the best of our knowledge, this is the first study to show that MuD exhibits an anti-apoptotic function following TRAIL treatment in astroglioma cells. Both overexpression and knockdown studies show that MuD may enable resistance against death stimuli including TRAIL. Moreover, the putative cleavage of MuD is likely to be an important step for the execution of apoptosis; MuD activation occurs prior to Bcl-2 cleavage upon TRAIL stimulation. In addition, our results demonstrate that Bid may be essential for the regulation of MuD expression and function at least in TRAIL-induced cell death and that MuD localizes predominantly in the ER and partly in mitochondria, then is decreased upon TRAIL stimulation (Figure 4h). In this regard, our findings suggest that MuD has an important role in TRAIL-mediated apoptotic signaling at the Bid and Bcl-2 junction.
