# Supplementary figures and images for: Characterization of MUDENG, a novel anti-apoptotic protein
Source: Oncogenesis. 2016 May 2;5(5):e221–. doi: 10.1038/oncsis.2016.30 (PMC4945747; doi:10.1038/oncsis.2016.30)

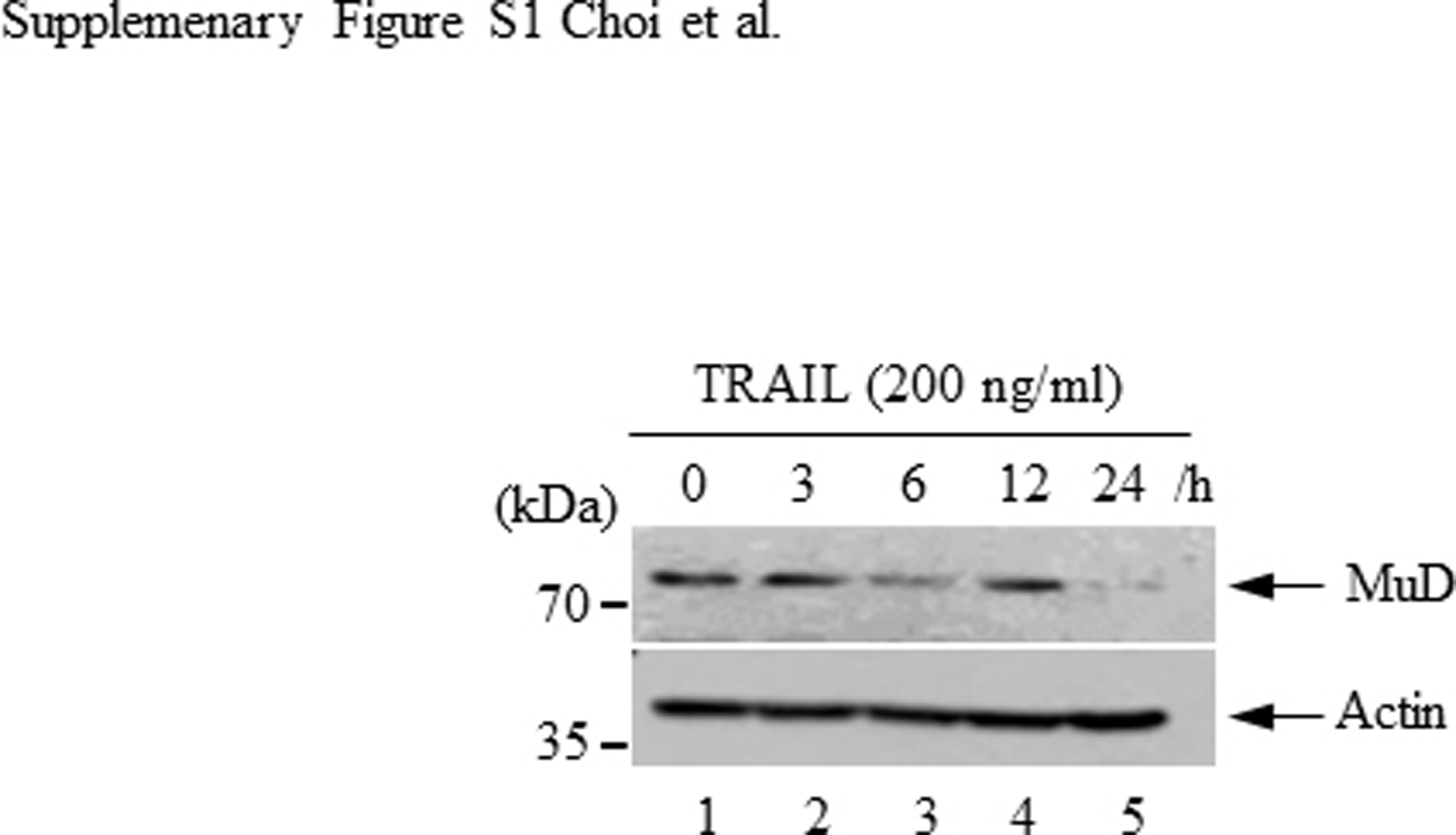

Supplement: Supplementary Figure 1 [file oncsis201630x1.tif]
