# Supplementary material for: Characterization of MUDENG, a novel anti-apoptotic protein
Source: Oncogenesis. 2016 May 2;5(5):e221–. doi: 10.1038/oncsis.2016.30 (PMC4945747; doi:10.1038/oncsis.2016.30)
Supplement: Supplementary Figure Legends [file oncsis201630x2.docx]

**Supplementary Figure 1**. T98G Cells (3 × 10^5^ cells/well) grown in 6-well plates were treated with TRAIL (200 ng/ml) for the indicated times (0–24 h). Cell lysates were separated by 10% SDS–PAGE then transferred onto a PVDF membrane. The pattern of MuD protein expression was analyzed using C22B3 MAb; β-actin served as a loading control.
